# Inflammatory status and severity of disease in dengue patients are associated with lipoprotein alterations

**DOI:** 10.1371/journal.pone.0214245

**Published:** 2019-03-22

**Authors:** Damariz Marin-Palma, Cherilyn M. Sirois, Silvio Urcuqui-Inchima, Juan C. Hernandez

**Affiliations:** 1 Infettare, Facultad de Medicina, Universidad Cooperativa de Colombia, Medellin, Colombia; 2 Grupo Inmunovirologia, Facultad de Medicina, Universidad de Antioquia, UdeA, Medellín, Colombia; 3 Department of Biology & Chemistry, Springfield College, Springfield, MA, United States of America; University of Hong Kong, HONG KONG

## Abstract

**Introduction:**

The triggering of severe dengue has been associated with an exacerbated inflammatory process characterized by the production of pro-inflammatory cytokines such as IL-1β/IL-18, which are the product of inflammasome activation. Furthermore, alteration in the levels of high-density (HDL) and low-density lipoproteins (LDL) has been observed; and HDL are known to have immunomodulatory properties, including the regulation of inflammasomes. While HDL would be expected to counteract hyperactivation of the inflammasome, the relationship between HDL and dengue severity, has not previously been explored.

**Methodology:**

We conducted a cross-sectional study of 30 patients with dengue and 39 healthy controls matched by sex and age. Lipid profile and levels of C-reactive protein were quantified. Serum levels of IL-1β, IL-6, IL-10, IL-18, and TNF-α, were assessed by ELISA. Expression of inflammasome-related genes in PBMC was quantified by qPCR.

**Results:**

Dengue patients presented an alteration in the parameters of the lipid profile, with a significant decrease in HDL levels, which was more pronounced in dengue patients with warning signs. Moreover, a decrease in the expression of the inflammasome-related genes NLRP1, NLRC4, caspase-1, IL-1β and IL-18 was observed, as well as an increase in serum levels of C-reactive protein and IL-10 in dengue patients versus healthy donors. Significant positive correlations between LDL levels and the relative expression of NLRP3, NLRC4, IL-1β and IL-18, were found.

**Conclusion:**

The results suggest that there is a relationship between the alteration of LDL and HDL with the imbalance in the inflammatory response, which could be associated with the severity of dengue.

## Introduction

Dengue virus (DENV) is responsible for one of the most prevalent infections in the world transmitted by arthropods, with nearly 100 million symptomatic infections annually. Dengue presents a wide spectrum of symptoms ranging from a feverish illness with headache, joint and muscle pain, up to severe manifestations such as multi-organ failure, hemorrhages and death [1]. Several studies have been conducted to identify the mechanisms involved in variations in the severity of the disease; antibody-dependent enhancement during a second infection [2, 3] and a cytokine storm [4], appear to be key factors. However, there are primary infections that also develop into severe dengue disease, which suggests the existence of additional mechanisms involved in the development of severe dengue symptoms. In this sense, it has been found that multiple baseline metabolic/immunologic parameters are related to the clinical state of the patients [5–8], including the characteristics of their serum lipid profile and a predisposition for an exacerbated inflammatory response [9]. Contradictory results have been reported for the lipid profile in dengue patients. It has been reported that patients with severe dengue present increased levels of triglycerides, very-low-density lipoprotein (VLDL) and high-density lipoproteins (HDL), as well as lower levels of total cholesterol and low-density lipoprotein (LDL), compared with other clinical presentations and healthy donors [10]. Other studies have described that triglycerides, total cholesterol and LDL have similar behavior to the healthy subjects, while HDL levels are lower in patients with severe dengue [11]. HDL are heterogeneous macromolecular complexes that transport cholesterol from peripheral tissues to the liver, for elimination [12, 13]. Of interest, here is that HDL exert pleiotropic effects on the immune system, such as reduction of inflammation and apoptosis [13–16]. Recently, it was reported that HDL regulate inflammasome activity [17]. The inflammasomes are cytosolic complexes involved in the maturation of the pro-forms of inflammatory cytokines of the IL-1 family, including IL-1β and IL-18 [18, 19]. Thacker *et al*. have reported that primary macrophages and human monocyte-like THP-1 cells produce lower levels of IL-1β in response to cholesterol crystals when pre-treated with HDL [17].

The involvement of inflammasome activation during viral infection [20], including DENV infection, is well established. Dengue patients have shown high levels of pro-inflammatory cytokines, especially those with severe clinical forms [21, 22]. Furthermore, it has been described that *in vitro* DENV-infection of macrophages induces an inflammatory response through activation of the NLRP3 inflammasome, leading to the release of IL-1β [23]. Likewise, it has been shown that platelets contribute to endothelial permeability through the activation of inflammasomes, specifically NLRP3, and the release of IL-1β, which seems to alter endothelial function [24].

Although there is evidence that both HDL levels and pro-inflammatory cytokines are altered during DENV infection [25], their specific relationship remains unknown. As lifestyle-based and pharmacological modulation of lipid profiles is commonly integrated into medical care, understanding a possible anti-inflammatory function of HDL or lipid profile changes during DENV infection could be helpful for managing dengue virus disease. Therefore, the aim of this work is to describe the characteristics of the lipid profile in dengue patients and to explore the association between HDL levels and inflammatory markers, particularly the inflammasome components, during dengue pathogenesis.

## Materials and methods

### Study population

Thirty dengue patients were included and classified as having dengue without warning signs (DNWS), dengue with warning signs (DWWS) or severe dengue (SD), based on the WHO classification, 2009 [26]. Patients with co-infections, history of statin treatment, cancer, pregnancy or mental health problems, were excluded. Likewise, 39 healthy individuals (controls) with a negative serum test result for dengue (*SD BIOLINE Dengue Duo rapid test*) were included, matched by age and sex, and from the geographical area to dengue-positive subjects. All subjects were adults, read and signed an informed consent approved by the ethics committee of the Universidad Cooperativa de Colombia, who also approved the whole study (0800–005). In addition, all research protocols were made according to the principles of the Declaration of Helsinki.

### Blood samples and processing

Plasma and serum were separate by centrifugation at 3500 rpm from approximately 20 mL of peripheral blood, which was obtained by venipuncture and collected in Vacutainer tubes (BD Diagnostics. Franklin Lakes, NJ 07417). The samples were stored at -70°C until use. For dengue diagnosis, the detection of NS1 antigen and IgM and IgG specific antibodies in serum were achieved by using the *SD BIOLINE Dengue Duo rapid test* (Dengue NS1 Ag and IgG/IgM; Alere, Australia). Peripheral blood mononuclear cells (PBMCs) were isolated using the gradient of density method with Ficoll-Histopaque (Sigma-Aldrich Chemical Co., St. Louis, MO) and were used for total RNA extraction, as described previously [27].

### Lipid profile and C-reactive protein (CRP) quantification

Triglycerides, total cholesterol and HDL levels were quantified in serum through colorimetric assays, using the Triglycerides, Total Cholesterol and Cholesterol HDL Direct kits (Biosystems Ref. 11828, 11506 and 11648, respectively; Costa Brava 30, Barcelona, Spain); the results were reported in mg/dL. Serum CRP was quantified by immunoturbidimetry in a certified clinical laboratory in Medellin- Colombia.

### Quantification of cytokines by ELISA

Serum levels of IL-1β, IL-6, IL-10, IL-18 y TNF-α were quantified by ELISA using commercial kits from BD Biosciences (Franklin Lakes, NJ) and eBioscience (Vienna, Austria), following the manufacturer’s instructions and in duplicate, as previously described in detail [27].

### mRNA quantification of the inflammasome-related genes

As previously reported in detail [27, 28], total RNA was isolated from PBMCs using the RNeasy Mini Kit (QIAGEN, Inc., Valencia, CA, USA), following the manufacturer’s recommendations. RNA concentration/purity was determined by spectrophotometry at 260/280 nm. cDNA synthesis was conducted with 230 ng of total RNA and using the Revertaid H Minus First Strand cDNA Synthesis Kit (Fermentas, Glen Burnie, MD, USA), following the manufacturer’s recommendations.

The mRNA of NLRP1, NLRP3, NLRC4, AIM2, ASC, caspase-1, IL-1β, and IL-18 was quantified by real time RT-PCR using the Maxima SYBR Green qPCR master mix (Fermentas). The specific amplification of β-actin (housekeeping gene) was used as a control. Amplification protocols were 39 cycles and standardized for each gene [28, 29], S1 Table. Real time RT-PCR analysis was conducted with the software CFX Manager, version: 1.5.534.0511 (Bio-Rad, Hercules, CA, USA).

### Statistical analysis

The statistical package GraphPad Prism 5.0 (San Diego, CA, USA) was used. Data were analyzed for normality and homoscedasticity using the Shapiro-Wilk and Levene tests. For the comparison between groups, the t-Student or Mann-Whitney test were applied for parametric and non-parametric data, respectively. For comparison between three or more groups, parametric ANOVA o Kruskal-Wallis tests were used; in case of a statistical association, post *hoc* tests (or multiple benchmarks) HDS of Tuckey o Dunn respectively, were carried out. Finally, for correlation analysis, Pearson or Spearman tests were applied to parametric or non-parametric data, respectively.

## Results

### Demographic data

Thirty dengue patients were included and classified according to their clinical presentation as DNWS, DWWS or SD and compared to 39 healthy controls, in a period between October 2015 and April 2017. Clinical and laboratory criteria used to classify the patients according to the severity of the disease included clinical symptoms such as fever, myalgia/arthralgia, headache, retro-ocular pain, asthenia, anorexia and presence of rash and pruritus. Additional symptoms like vomiting, abdominal pain, diarrhea, cough, sore throat, dizziness, somnolence, lethargy, irritability, and convulsions were also considered. Hemorrhagic signs, including petechiae, purpura/ecchymosis, epistaxis, hematemesis/melena, and hematuria were included. As well as, plasma leakage and organ dysfunction manifestations. Finally, the laboratory parameters electrolytes, leukocytes and platelets counts, hematocrit, and transaminases were assessed. Major demographic and clinical data are presented in Table 1. It is noteworthy that the majority of patients (92%) presented thrombocytopenia as characterized by a platelet count below the reference value of 150.000 platelets/μL. Days of evolution of dengue disease had a median of around 6 days for all the groups; and the number of patients presenting with primary (n = 13) and secondary (n = 17) DENV infections was comparable.

**Table 1:** Clinical and demographic information.

	Group			
	Controls (n = 39)	Dengue patients		
		DNWS (n = 8)	DWWS (n = 18)	SD (n = 4)
Women : Men	16 : 23	1 : 7	8 : 10	3 : 1
Age in years: Median (IQR)	35 (26)	42 (22.7)	29.5 (32.5)	46.5 (44.0)
Days of evolution: Median (IQR)		5.0 (2.8)	6.0 (1.5)	6.5 (2.5)
% Hematocrit: Median (IQR)		46.2 (11.0)	42.9 (4.7)	41.6 (5.0)
Platelets/μL: Median (IQR)		124,000 (126,000)	73,000 (57,000)	44,500 (112,750)
AST in IU/mL: Median (IQR)	RR (15–37)	74 (138.5)	137 (140)	161.5 (745.3)
ALT en IU/mL: Median (IQR)	RR (30–65)	43 (72.5)	122 (124.5)	93 (583)
1° infection: 2° infection		4:4	8:10	1:3
Hepatomegaly / Splenomegaly (%)		0	5.6	50
Petechiae (%)		37.8	44.4	75

RR: Reference range, IQR: Interquartile range, IU: International unit, AST: Aspartate transaminase, ALT: Alanine transaminase, DNWS: Dengue with no warning signs, DWWS: Dengue with warning signs and SD: Severe dengue.

### Dengue patients have an altered standard lipid profile

**Fig 1:**
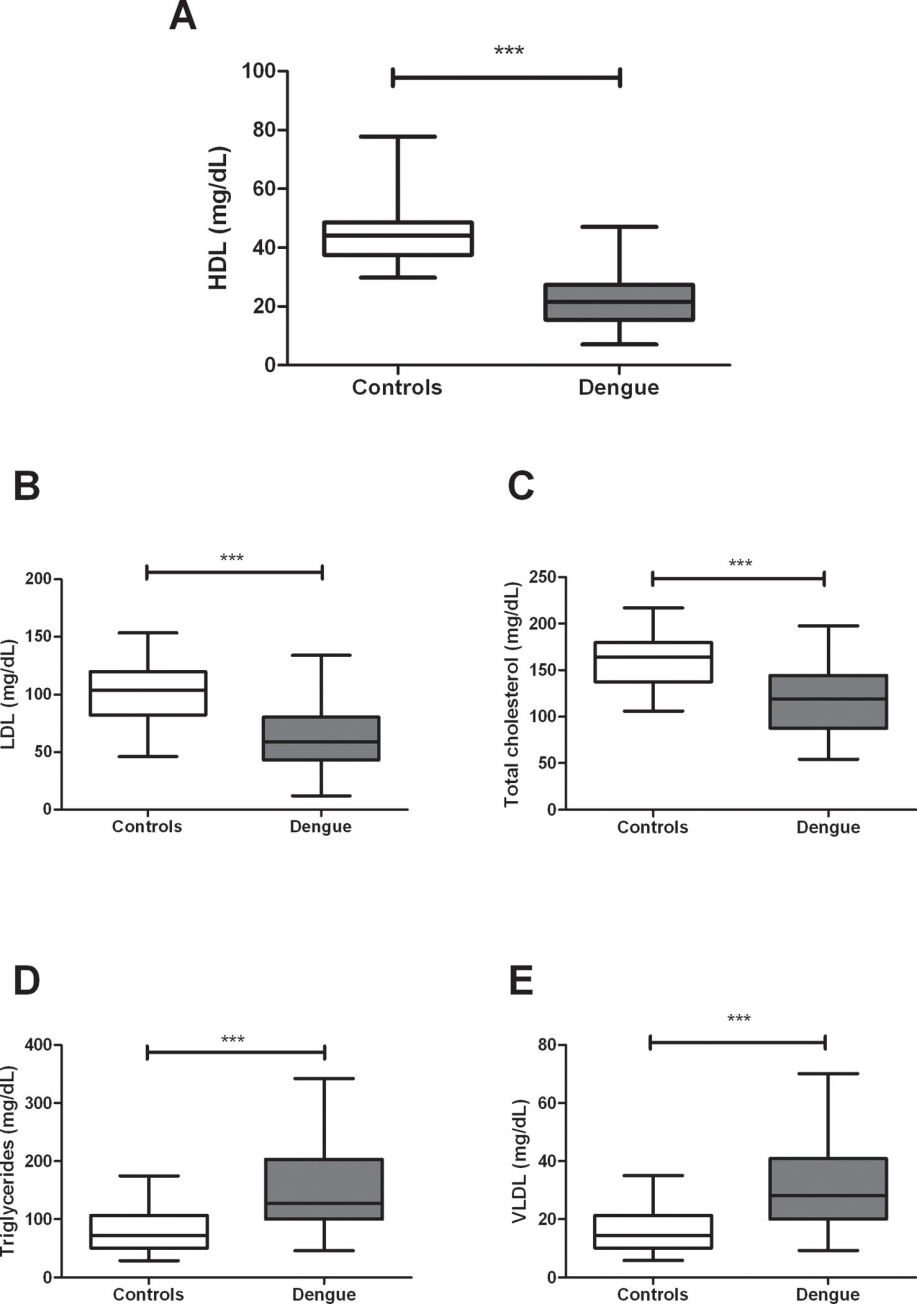
Serum lipid profile in individuals with dengue differs from that of healthy controls. (A) HDL, (B) LDL, (C) Total cholesterol, (D) Triglycerides and (E) VLDL were quantified by duplicated, using a colorimetric assay. The results are presented as medians with maximum and minimum values (box and whisker plot). Statistical comparison was made using the Mann-Whitney U test with a confidence level of 95%. Significant differences are represented at the top of the figure (***p< 0.001).

**Fig 2:**
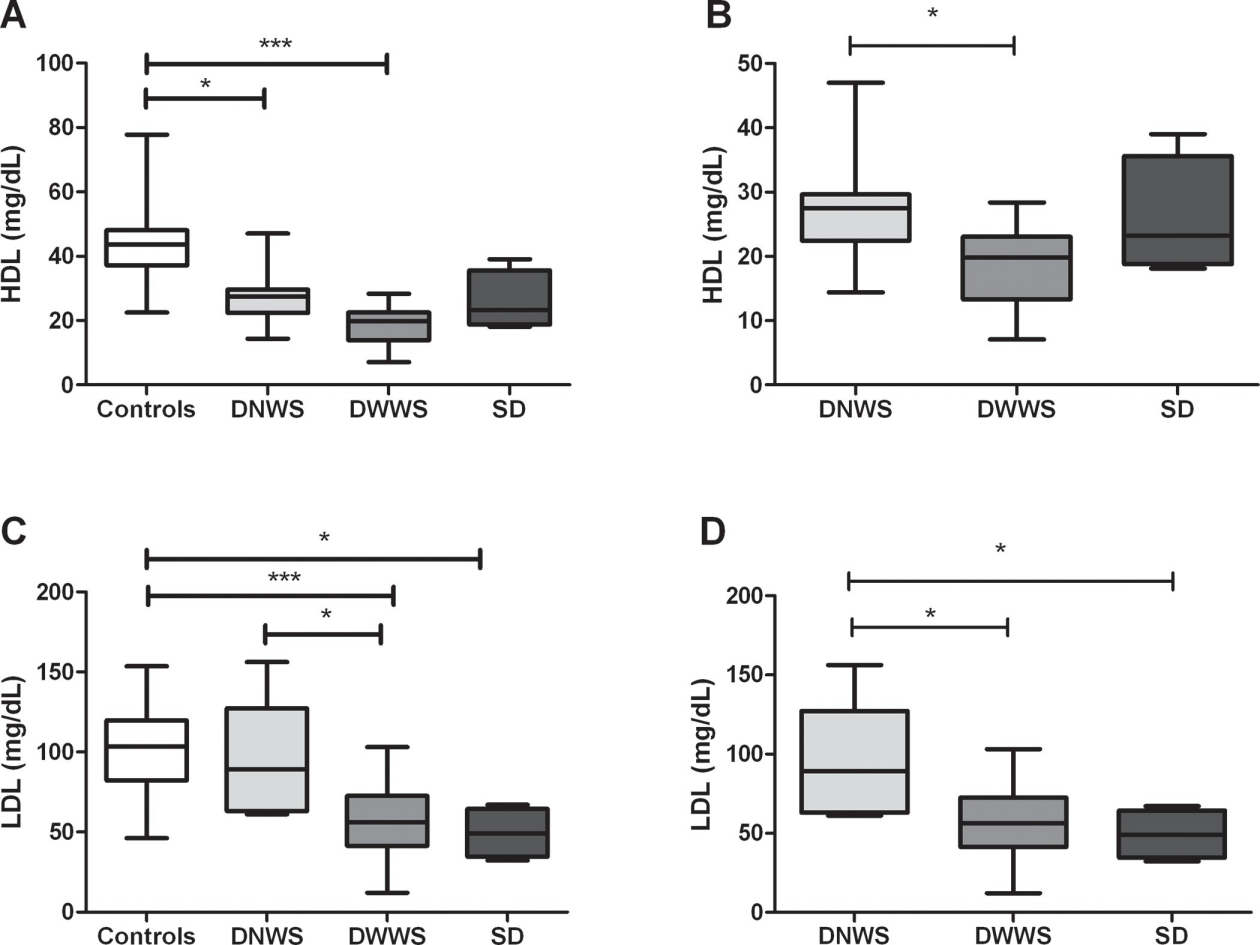
Serum HDL and LDL levels from dengue patients according to clinical classification. HDL (A and B) and LDL (C and D) levels were assessed by duplicated; in the serum of individuals classified has having dengue with no warning signs (DNWS), dengue with warning signs (DWWS) or severe dengue (SD). The results are presented as medians with maximum and minimum values (box and whisker plot). Statistical comparison was made using the Kruskal-Wallis test with a confidence level of 95% and post *hoc* tests (or multiple benchmarks) HDS of Dunn were applied. Significant differences are represented at the top of the figure (*p<0.05; ***p<0.001).

During dengue infection, patients have previously been shown to have multiple metabolic alterations associated with the acute inflammatory response, including the lipid profile [10]. In our study population, dengue patients had significantly lower HDL levels (*p*<0.0001, Fig 1A) compared to healthy controls. Similarly, lower LDL (p = 0.0002; Fig 1B) and total cholesterol (*p* <0.0001; Fig 1C) levels and higher triglycerides and VLDL levels (*p* < 0.0001; Fig 1D and 1E, respectively) were observed. The difference in HDL levels in dengue patients versus controls was maintained even when patients were distinguished by clinical disease severity (DNWS, DWWS and SD; Fig 2A). Moreover, when HDL levels were compared between the groups of dengue patients, those with more severe clinical disease (DWWS group) had lower HDL levels than those with no clinical warning signs (DNWS group; *p*<0.05; Fig 2B), but no differences were found when comparing with SD group. Similar results were observed with respect to the LDL levels in dengue patients according to their clinical stages (Fig 2C and 2D).

### Dengue patients show decreased expression of inflammasomes-related genes

**Fig 3:**
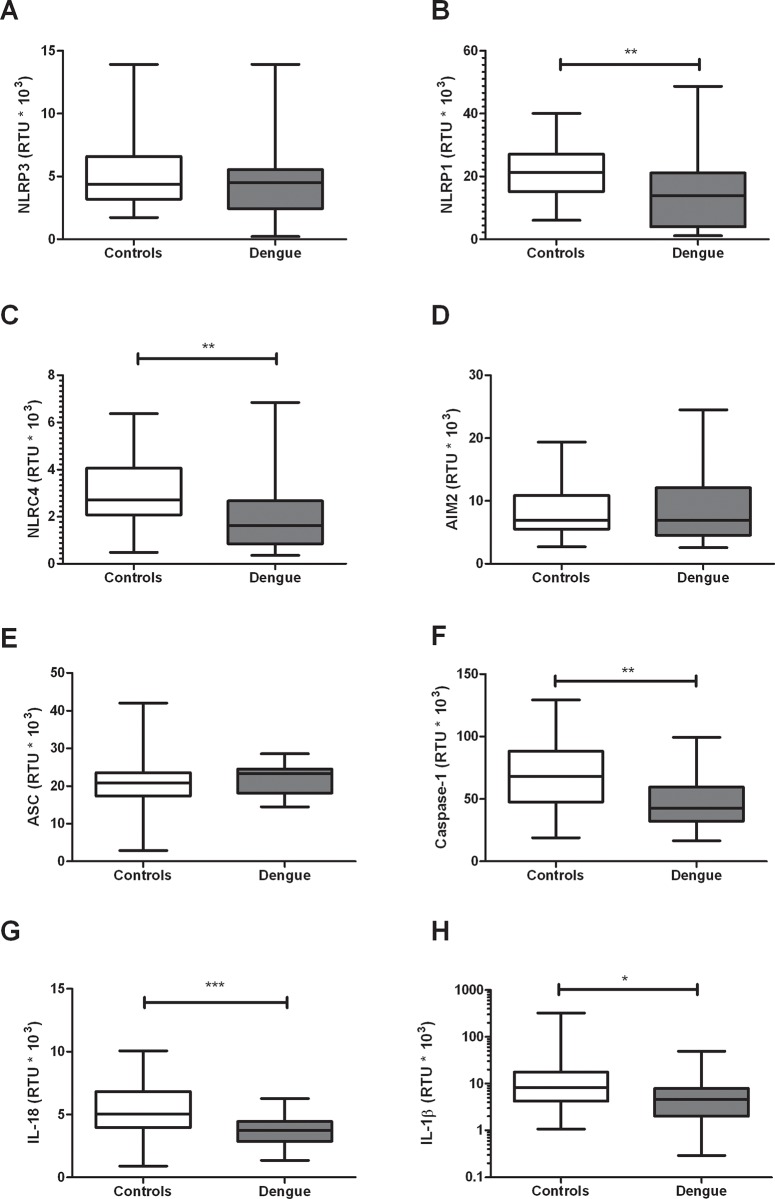
Inflammasome-related genes expression in dengue patients. The expression of (A) NLRP3, (B) NLRP1, (C) NLRC4, (D) AIM2, (E) ASC, (F) caspase-1, (G) IL-18 and (H) IL-1β was evaluated by real time PCR in PBMCs (by duplicates) from dengue patients and healthy controls. The β-actin gene was used as a constituent gene to normalize the RNA content. The results are presented as medians with maximum and minimum values (box and whisker plot). Statistical comparison was made using the Mann-Whitney U tests with a confidence level of 95%. Significant differences are represented at the top of the figure (*p< 0.05, **p < 0.01; ***p < 0.001).

**Fig 4:**
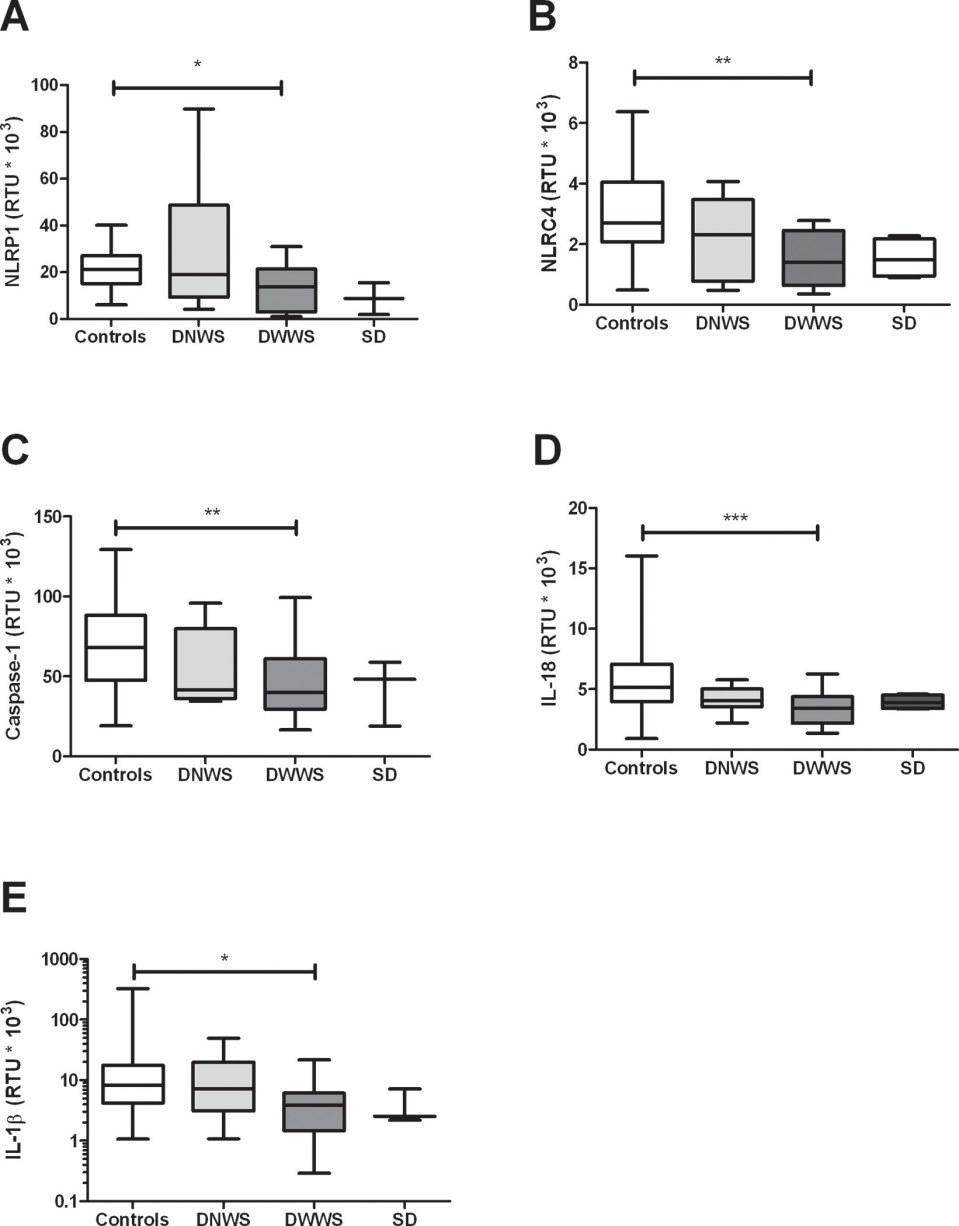
Inflammasome-related genes expression in dengue patients, according to clinical classification. The expression of (A) NLRP1, (B) NLRC4, (C) caspase-1, (D) IL-18 and (E) IL-1β in PBMCs was quantified by real time PCR by duplicated. The β-actin gene was used as a constitutive gene to normalize the mRNA content. The results are presented as medians with maximum and minimum values (box and whisker plot). Statistical comparison was made using the Kruskal-Wallis test with a confidence level of 95% and post *hoc* tests (or multiple benchmarks) HDS of Dunn, were applied. Significant differences are represented at the top of the figure (*p < 0.05; **p < 0.01; ***p < 0.001).

The NLRP3 inflammasome has been shown to be activated during DENV infection [23]. Here we found that the mRNA levels of NLRP1 (*p* = 0.0032), NLRC4 (*p* = 0.0026), caspase-1 (*p* = 0.0005), IL-18 (*p*< 0.0001) and IL-1β (*p* = 0.0179), were lower in dengue patients compared with healthy controls (Fig 3), however differences between NLRP3, AIM2 and ASC were not found. When inflammasome-related genes expression was evaluated according to the clinical stages of dengue, a statistically significant decrease was found in the mRNA of NLRP1 (*p*< 0.05), NLRC4 (*p*< 0.01), caspase-1 (*p*< 0.01) IL-18 (*p*< 0.0001) and IL-1β (*p*< 0.05) in DWWS patients, compared with healthy controls (Fig 4).

### Dengue patients express higher levels of IL-10 and C-reactive protein

**Fig 5:**
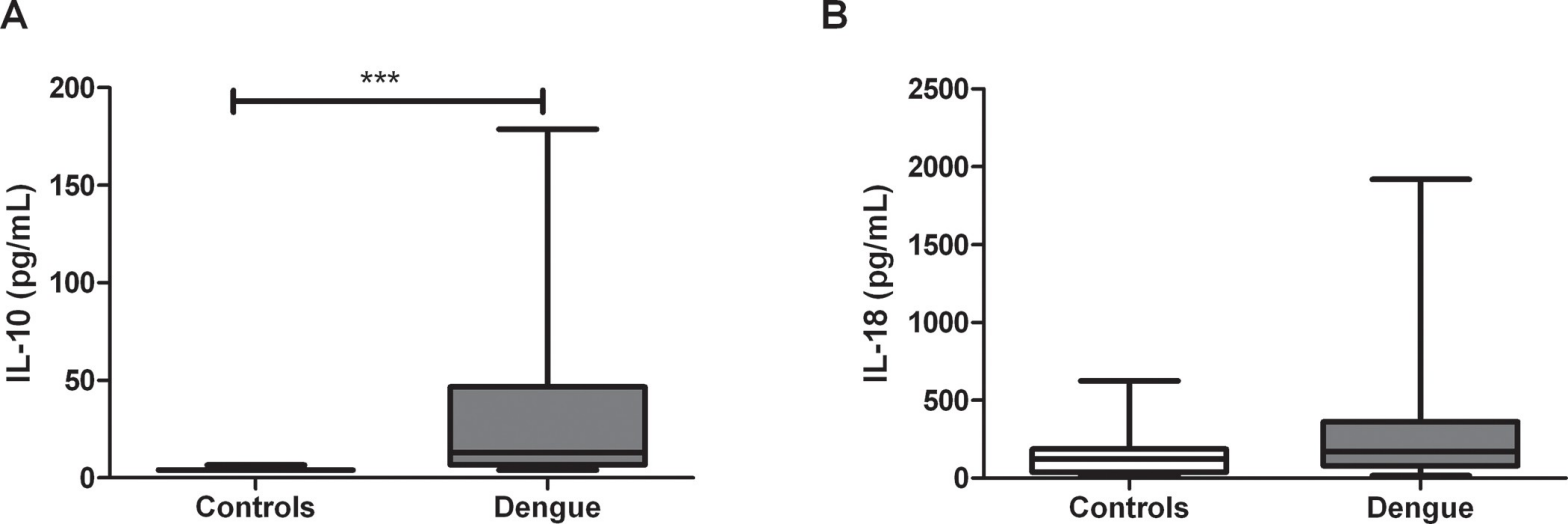
Serum levels of IL-10 and IL-18 in dengue patients. Serum levels of (A) IL-10 and (B) IL-18 were quantified by ELISA (in duplicates). The results are presented as medians with maximum and minimum values (box and whisker plot). The statistical comparison was made using the Mann Whitney U test with a confidence level of 95%. Significant differences are represented at the top of the figure (***p < 0.001).

The next step was to quantify the serum levels of IL-1β, IL-6, IL-18, IL-10, TNF-α and CRP, as indicators of the inflammatory status in the dengue patients. A significant increase in IL-10 levels from dengue patients was observed in comparison to healthy controls (*p*< 0.0001; Fig 5A). However, the IL-18 levels were similar in individuals with dengue versus healthy controls (Fig 5B). No detectable serum levels of IL-1β, IL-6 and TNF-α, were observed.

**Fig 6:**
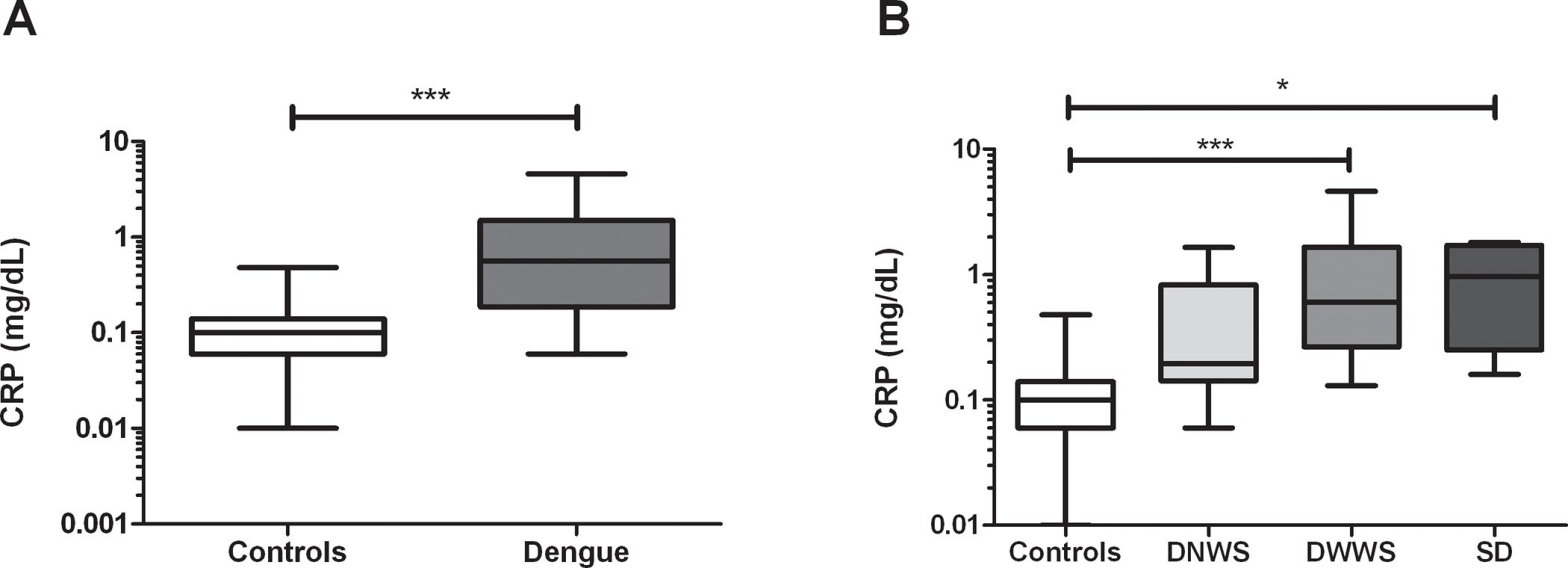
Serum C-reactive protein levels in dengue patients. Serum levels of CRP were quantified in dengue patients (A) total dengue patients or (B) dengue patients according to the clinical stages and healthy controls, using a colorimetric assay The results are presented as medians with maximum and minimum values (box and whisker plot). The statistical comparison was made using the Mann Whitney U and Kruskal-Wallis tests with a confidence level of 95% and post *hoc* tests (or multiple benchmarks) HDS of Dunn, were applied. Significant differences are represented at the top of the figure (*p<0.05 and ***p < 0.001).

Likewise, an increase in serum CRP levels was found in dengue patients compared to healthy controls (*p*< 0.0001, Fig 6A), which was more evident for DWWS and SD patients (*p*< 0.0001 and p< 0.05 respectively; Fig 6B).

### LDL levels are positively correlated with inflammasomes

**Fig 7:**
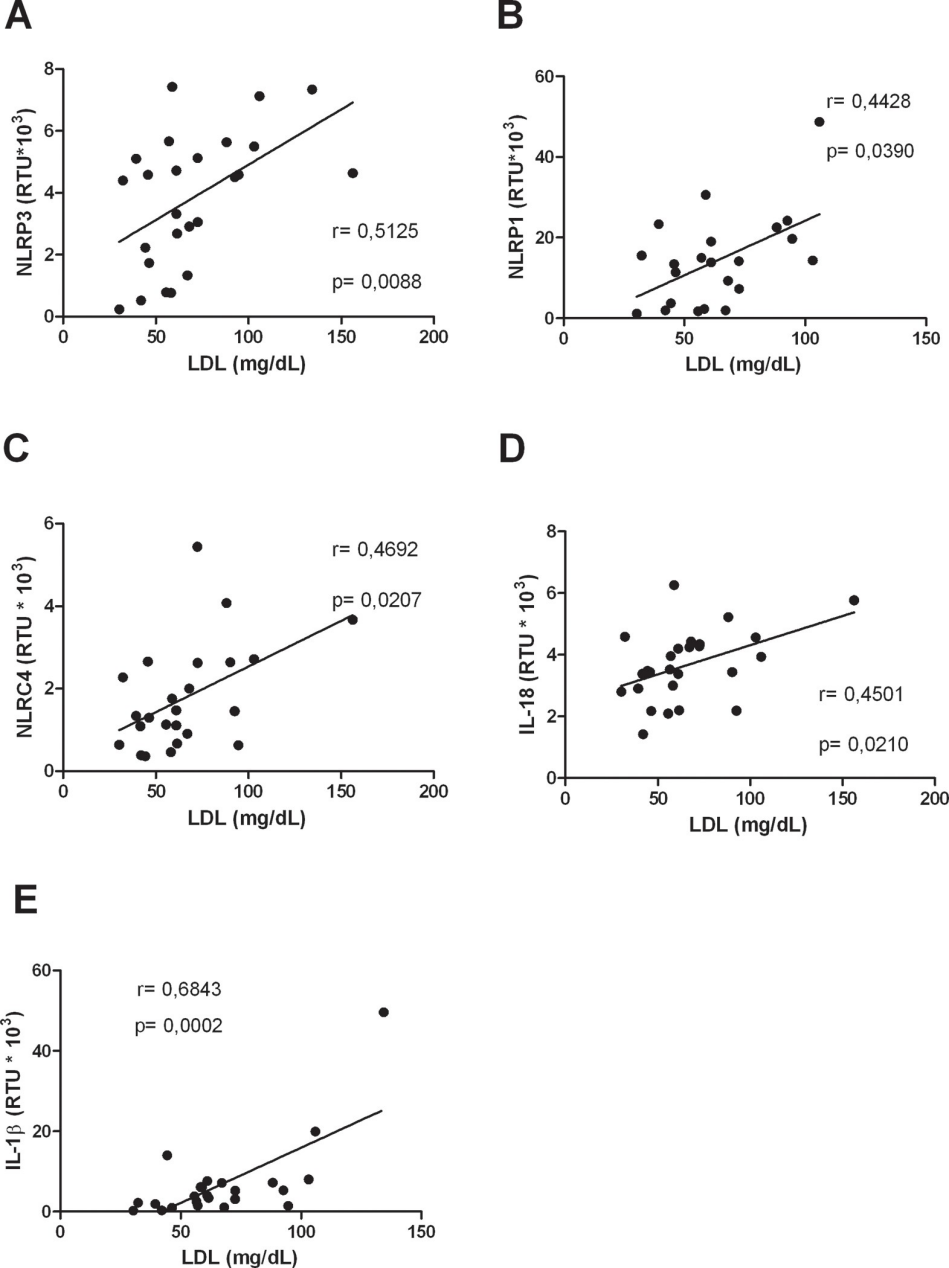
LDL levels are correlated with inflammasome-related genes. Correlation between LDL levels and (A) NLRP3, (B) NLRP1, (C) NLRC4, (D) IL-18 and (E) IL-1β. Correlations between variables were assessed using the Spearman test. The *r*-value and *p*-value are indicated in each figure. A *p*-value of less than 0.05 was considered a significant correlation.

Previously it was reported that LDL have the ability to induce the production of IL-1β, through the NF-κB and caspase-1 pathways in macrophages [30]. Since our results showed an alteration in lipoproteins levels in dengue patients compared to controls, we proceeded to analyze whether HDL and LDL levels might statistically correlate with inflammasome-related genes expression. While no significant correlations were observed for HDL (S1 Fig); positive correlations were found between LDL and NLRP3, NLRP1, NLRC4, IL-18 and IL-1β (Fig 7).

## Discussion

At present, several strategies are employed to modulate blood lipoprotein levels aiming to reduce the risk of cardiovascular disease. Strategies based on a healthy lifestyle, such as regular exercise and a low-fat diet, and the use of statins, are the most common. However, the impact that HDL may have on other diseases, such as viral infections, is just beginning to be studied as evidence of its immunomodulatory effects emerges. Here we present evidence supporting the hypothesis that concurrent changes in the serum lipid profile and inflammasome-mediated cytokine production may be mechanistically relevant in the progression of DENV infection to clinically severe forms of dengue. While previous reports regarding the HDL levels of dengue patients are contrasting [9, 10], we found that patients with dengue had lower levels of HDL, LDL and total cholesterol compared to the healthy controls, similar to that reported by Biswas *et al*. [9]. When the comparison was made according to the clinical stages, the DWWS patients had lower levels of HDL and LDL than those DNWS patients. These results suggest that patients with lower HDL and LDL levels may be progressing to the life-threatening forms of the disease. However, it is noteworthy that our severe dengue (SD) patients did not show a clear behavior as the DWWS patients. The discrepancies observed regarding the relationship between HDL and the severe forms of dengue may be due to differences in the study design and the population included. While the study carried out by Duran et al. [10] had a cross-sectional design in a population between 2 and 66 years and sampling at the acute phase, the study by Biswas et al. [9] was longitudinal, on pediatric patients. In this sense, the difference in age could influence the lipid profile, and the behavior of the studied markers in response to dengue infection may be also different. Moreover, it is important to mention that although we did a cross sectional study, the lipoprotein levels appear to correlate with the clinical phase of the infection. Taken together these observations and the previous report of increased HDL in patients with SD [10, 25], this suggests that the relationship between lipid levels and clinical severity is not strictly linear, and that further studies (especially with longitudinal designs) are needed to clearly define the mechanistic relationship between serum lipids and dengue pathology, according to age and clinical phase.

Several studies provide context for understanding the participation of cholesterol-containing lipoproteins like HDL and LDL in DENV infection. Puerta-Guardo *et al*. report altering the formation of plasma membrane lipid rafts by eliminating cholesterol reduces DENV infection of host cells [31]. In addition, it has been reported that there is less production of dengue viral particles in cells treated with lovastatin, a drug that decreases the production of cholesterol [32]. Likewise, the elimination of cholesterol from the viral particles reduces their infectivity by affecting the fusion with the endosomal membranes, thus blocking the replication cycle [33]. In light of these findings, it is interesting to consider that HDL has also been shown to modulate the cholesterol content of the lipid rafts, mainly through the ABCA1 and ABCG1 transporter proteins [34]. Considering that DWWS patients showed lower levels of HDL, it is interesting to speculate that the decrease in this lipoprotein during DENV infection could lead to reduced cholesterol flow in the lipid rafts; thus favoring a better production of DENV particles due to a higher level of cholesterol in the cells, and that this contributes to greater severity of the disease.

While serum lipids may be able to directly affect DENV virology, the interrelation of lipids with the activation and maintenance of inflammation is likely to also play a role in dengue pathogenesis. DENV infection induces temporal changes in the lipidome and serum metabolome, thus modulating the development and maintenance of inflammatory responses [35]. Previous *in vitro* studies have reported that DENV can promote the expression of IL-1β and IL-18 in macrophages through the activation of the NLRP3 inflammasome [23]. In addition, infection of platelets with DENV leads to the assembly of the NLRP3 inflammasome, activation of caspase-1, and the production of IL-1β, which is secreted in microparticles and can promote increased vascular permeability [24], a phenomenon associated with the severity of the disease. It has also been found that other arboviruses, such as Chikungunya and West Nile virus, have the ability to activate inflammasomes and induce the expression of AIM2 [36]. However, the present study found a decrease in some inflammasome components in patients with dengue, especially in DWWS patients. This may be due to the clinical stages of the patients (critical and convalescent), in which the inflammatory response could be negatively regulated. In this concern, it is important to note that an increase in IL-10 was found in dengue patients; this anti-inflammatory cytokine is produced late in infection [37], has immunosuppressive functions, and is associated with greater severity of infection [38, 39]. So, it is possible that inflammasome activation is established at the beginning of viral infection and strong production of pro-inflammatory cytokines contributes to immune-pathogenesis and clinical disease outcome. However, in the later phase of the infection, regulatory mechanisms (evidenced by IL-10) are activated and downregulate expression of inflammasome components, as observed in this study.

In corroboration of this logic, previous studies have reported that dengue patients show a decrease in IL-1β levels, which return to baseline levels after the recovery phase [40]. Moreover, this decrease in IL-1β is more significant in patients with hospitalization requirements compared to those who do not require it [41], suggesting that patients with lower IL-1β production present greater severity of the disease. It could be important to consider the clinical classification algorithm used in this study, in comparison with other reports using the previous classification strategies. The hypothesis that patients with DWWS have decreased levels of IL-1β could be considered, but given the lack of infection control, the levels increase again. This could explain our results showing that DWWS patients exhibited less expression of some inflammasome components. And also, the results reported by other authors were found increased expression of IL-1β in patients with severe dengue [21], as well as other pro-inflammatory cytokines [42, 43]. However, is necessary considering other differences in the study design, including the disease stage when the samples were taken. For this reason, it would be important to carry out longitudinal studies to evaluate the inflammasome activation and the production of cytokines during the different phases of the disease, in order to elucidate the production dynamics of these molecules and their association with the clinical outcomes.

While we found a decrease in the expression of inflammasome-related genes in dengue patients, we also found increased levels of the inflammatory marker CRP, and this was higher in DWWS patients, suggesting a residual inflammatory process. This is in accord with the previously reported observation that patients with dengue who had plasma loss (a characteristic of severe disease) presented higher levels of CRP [44]. Taken together, these data suggest that the inflammatory response during dengue infection is complex, and that the balance and dynamics between pro-inflammatory molecules can promote the serious forms of the disease.

Although HDL can negatively modulate inflammasome activation [17], in the present study no correlations were found between HDL and the expression of inflammasome-related genes in dengue patients. However, a decrease in HDL levels may lead to an alteration in HDL’s known anti-inflammatory activities, thus inducing an imbalance in the inflammatory response and consequently greater pathogenesis of the disease.

On the other hand, a positive correlation was found between LDL and some inflammasome components. Our findings indicated that dengue patients with higher levels of LDL also showed higher content of mRNA from inflammasome-related genes. This is noteworthy because oxidized LDL can promote the activation of the NLRP3 inflammasome [45]. In addition, DENV infection favors the cellular uptake of LDL [46], leading to an increase in intracellular cholesterol and subsequent formation of cholesterol crystals [47]; this, in turn, could be promoting the activation of the inflammasomes [48] and contributing to the inflammatory response involved in the resolution of the infection. It is possible that this relationship proceeds during the initial stage of the disease; nevertheless, continuous LDL uptake leads to decrease of this lipoprotein, as observed in the present study.

## Conclusion

The development of an effective immune response against infection by DENV is necessary for a favorable clinical outcome. However, the regulation of this response is equally fundamental to prevent tissue damage and severe disease symptoms. The results presented here indicate a relationship between serum lipoproteins and the inflammatory response during DENV infection. Specifically, the presented data support a relationship between LDL and inflammasome-related genes, which could modulate the dengue disease outcome. The present findings must be interpreted in the context of the time of disease progression in the studied patients, and suggest the need for longitudinal studies that will be capable of more completely assessing the dynamics of individual lipids and inflammation markers, as well as their correlations over the course of disease progression.

## Supporting information

S1 Fig Correlations between HDL and inflammasome-related genes. Correlation between HDL levels and (A) NLRP3, (B) NLRP1, (C) NLRC4, (D) AIM2, (E) Caspase-1, (F) ASC, (G) IL-18 and (H) IL-1β were assessed by the Spearman test. The *r*-value and *p*-value are indicated in the figure. A *p*-value lower than 0.05 was considered a significant correlation.
(TIF)

S1 Table Sequence of the primers. (DOCX)

## References

[1] (TDR) WHOWatSPfRaTiTD. Dengue guidelines for diagnosis, treatment, prevention and control. 2009. 23762963

[2] Dejnirattisai W, Jumnainsong A, Onsirisakul N, Fitton P, Vasanawathana S, Limpitikul W, et al Cross-reacting antibodies enhance dengue virus infection in humans. Science. 2010;328(5979):745–8. 10.1126/science.1185181 20448183 PMC3837288

[3] Ng JK, Zhang SL, Tan HC, Yan B, Martinez JM, Tan WY, et al First experimental in vivo model of enhanced dengue disease severity through maternally acquired heterotypic dengue antibodies. PLoS pathogens. 2014;10(4):e1004031 10.1371/journal.ppat.1004031 24699622 PMC3974839

[4] Kuczera D, Assolini JP, Tomiotto-Pellissier F, Pavanelli WR, Silveira GF. Highlights for Dengue Immunopathogenesis: Antibody-Dependent Enhancement, Cytokine Storm, and Beyond. Journal of interferon & cytokine research: the official journal of the International Society for Interferon and Cytokine Research. 2018;38(2):69–80. 10.1089/jir.2017.0037 29443656

[5] Bethell DB, Flobbe K, Cao XT, Day NP, Pham TP, Buurman WA, et al Pathophysiologic and prognostic role of cytokines in dengue hemorrhagic fever. The Journal of infectious diseases. 1998;177(3):778–82 9498463 10.1086/517807

[6] Lee WL, Liles WC. Endothelial activation, dysfunction and permeability during severe infections. Current opinion in hematology. 2011;18(3):191–6. 10.1097/MOH.0b013e328345a3d1 21423012

[7] Nascimento EJ, Braga-Neto U, Calzavara-Silva CE, Gomes AL, Abath FG, Brito CA, et al Gene expression profiling during early acute febrile stage of dengue infection can predict the disease outcome. PLoS One. 2009;4(11):e7892 10.1371/journal.pone.0007892 19936257 PMC2775946

[8] Nascimento EJ, Silva AM, Cordeiro MT, Brito CA, Gil LH, Braga-Neto U, et al Alternative complement pathway deregulation is correlated with dengue severity. PLoS One. 2009;4(8):e6782 10.1371/journal.pone.0006782 19707565 PMC2728508

[9] Biswas HH, Gordon A, Nunez A, Perez MA, Balmaseda A, Harris E. Lower Low-Density Lipoprotein Cholesterol Levels Are Associated with Severe Dengue Outcome. PLoS neglected tropical diseases. 2015;9(9):e0003904 10.1371/journal.pntd.0003904 26334914 PMC4559460

[10] Duran A, Carrero R, Parra B, Gonzalez A, Delgado L, Mosquera J, et al Association of lipid profile alterations with severe forms of dengue in humans. Archives of virology. 2015;160(7):1687–92. 10.1007/s00705-015-2433-z 25936955

[11] van Gorp EC, Suharti C, Mairuhu AT, Dolmans WM, van Der Ven J, Demacker PN, et al Changes in the plasma lipid profile as a potential predictor of clinical outcome in dengue hemorrhagic fever. Clinical infectious diseases: an official publication of the Infectious Diseases Society of America. 2002;34(8):1150–3. 10.1086/339539 11915007

[12] Asztalos BF, de la Llera-Moya M, Dallal GE, Horvath KV, Schaefer EJ, Rothblat GH. Differential effects of HDL subpopulations on cellular ABCA1- and SR-BI-mediated cholesterol efflux. J Lipid Res. 2005;46(10):2246–53. 10.1194/jlr.M500187-JLR200 16061948

[13] Marín-Palma D TN, Urcuqui-Inchima S, Hernández JC. Inflamación y respuesta inmune innata: Participación de las lipoproteínas de alta densidad. IATREIA. 2017 Oct-Dic;30(4):423–35. 10.17533/udea.iatreia.v30n4a06

[14] Uittenbogaard A, Shaul PW, Yuhanna IS, Blair A, Smart EJ. High density lipoprotein prevents oxidized low density lipoprotein-induced inhibition of endothelial nitric-oxide synthase localization and activation in caveolae. J Biol Chem. 2000;275(15):11278–83 10753938 10.1074/jbc.275.15.11278

[15] Kameda T, Ohkawa R, Yano K, Usami Y, Miyazaki A, Matsuda K, et al Effects of Myeloperoxidase-Induced Oxidation on Antiatherogenic Functions of High-Density Lipoprotein. Journal of lipids. 2015;2015:592594 10.1155/2015/592594 26257958 PMC4516847

[16] Nofer JR, Levkau B, Wolinska I, Junker R, Fobker M, von Eckardstein A, et al Suppression of endothelial cell apoptosis by high density lipoproteins (HDL) and HDL-associated lysosphingolipids. J Biol Chem. 2001;276(37):34480–5. 10.1074/jbc.M103782200 11432865

[17] Thacker SG, Zarzour A, Chen Y, Alcicek MS, Freeman LA, Sviridov DO, et al High-density lipoprotein reduces inflammation from cholesterol crystals by inhibiting inflammasome activation. Immunology. 2016;149(3):306–19. 10.1111/imm.12638 27329564 PMC5046053

[18] Broz P, Dixit VM. Inflammasomes: mechanism of assembly, regulation and signalling. Nature reviews Immunology. 2016;16(7):407–20. 10.1038/nri.2016.58 27291964

[19] Im H, Ammit AJ. The NLRP3 inflammasome: role in airway inflammation. Clinical and experimental allergy: journal of the British Society for Allergy and Clinical Immunology. 2014;44(2):160–72. 10.1111/cea.12206 24118105

[20] Feria MG, Taborda NA, Hernandez JC, Rugeles MT. HIV replication is associated to inflammasomes activation, IL-1beta, IL-18 and caspase-1 expression in GALT and peripheral blood. PLoS One. 2018;13(4):e0192845 10.1371/journal.pone.0192845 29672590 PMC5909617

[21] Bozza FA, Cruz OG, Zagne SM, Azeredo EL, Nogueira RM, Assis EF, et al Multiplex cytokine profile from dengue patients: MIP-1beta and IFN-gamma as predictive factors for severity. BMC infectious diseases. 2008;8:86 10.1186/1471-2334-8-86 18578883 PMC2474613

[22] Mustafa AS, Elbishbishi EA, Agarwal R, Chaturvedi UC. Elevated levels of interleukin-13 and IL-18 in patients with dengue hemorrhagic fever. FEMS immunology and medical microbiology. 2001;30(3):229–33 10.1111/j.1574-695X.2001.tb01575.x 11335143

[23] Wu MF, Chen ST, Yang AH, Lin WW, Lin YL, Chen NJ, et al CLEC5A is critical for dengue virus-induced inflammasome activation in human macrophages. Blood. 2013;121(1):95–106. 10.1182/blood-2012-05-430090 23152543

[24] Hottz ED, Lopes JF, Freitas C, Valls-de-Souza R, Oliveira MF, Bozza MT, et al Platelets mediate increased endothelium permeability in dengue through NLRP3-inflammasome activation. Blood. 2013;122(20):3405–14. 10.1182/blood-2013-05-504449 24009231 PMC3829114

[25] Barrientos-Arenas E, Henao-García V, Giraldo DM, Cardona MM, Urcuqui-Inchima S, Castaño JC, et al Modulación de los niveles de lipoproteínas de alta densidad y las citoquinas IL-1β e IL-6 en pacientes con dengue. Revista peruana de medicina experimental y salud publica. 2018;35 (1):15–24. 10.17843/rpmesp.2018.351.3568 29924262

[26] Diseases WHOSPfRaTiT. Dengue Guidelines for Diagnosis, Treatment, Prevention and Control. 2009. Available from: http://www.who.int/tdr/publications/documents/dengue-diagnosis.pdf 23762963

[27] Marin-Palma D, Castro GA, Cardona-Arias JA, Urcuqui-Inchima S, Hernandez JC. Lower High-Density Lipoproteins Levels During Human Immunodeficiency Virus Type 1 Infection Are Associated With Increased Inflammatory Markers and Disease Progression. Frontiers in immunology. 2018;9:1350 10.3389/fimmu.2018.01350 29963050 PMC6010517

[28] Gomez DM, Urcuqui-Inchima S, Hernandez JC. Silica nanoparticles induce NLRP3 inflammasome activation in human primary immune cells. Innate Immun. 2017;23(8):697–708. 10.1177/1753425917738331 29113588

[29] Hernandez JC, Giraldo DM, Paul S, Urcuqui-Inchima S. Involvement of neutrophil hyporesponse and the role of Toll-like receptors in human immunodeficiency virus 1 protection. PLoS One. 2015;10(3):e0119844 10.1371/journal.pone.0119844 25785697 PMC4364960

[30] Estruch M, Rajamaki K, Sanchez-Quesada JL, Kovanen PT, Oorni K, Benitez S, et al Electronegative LDL induces priming and inflammasome activation leading to IL-1beta release in human monocytes and macrophages. Biochimica et biophysica acta. 2015;1851(11):1442–9. 10.1016/j.bbalip.2015.08.009 26327597

[31] Garcia Cordero J, Leon Juarez M, Gonzalez YMJA, Cedillo Barron L, Gutierrez Castaneda B. Caveolin-1 in lipid rafts interacts with dengue virus NS3 during polyprotein processing and replication in HMEC-1 cells. PLoS One. 2014;9(3):e90704 10.1371/journal.pone.0090704 24643062 PMC3958351

[32] Martinez-Gutierrez M, Castellanos JE, Gallego-Gomez JC. Statins reduce dengue virus production via decreased virion assembly. Intervirology. 2011;54(4):202–16. 10.1159/000321892 21293097

[33] Carro AC, Damonte EB. Requirement of cholesterol in the viral envelope for dengue virus infection. Virus research. 2013;174(1–2):78–87. 10.1016/j.virusres.2013.03.005 23517753

[34] Murphy AJ, Woollard KJ, Hoang A, Mukhamedova N, Stirzaker RA, McCormick SP, et al High-density lipoprotein reduces the human monocyte inflammatory response. Arterioscler Thromb Vasc Biol. 2008;28(11):2071–7. 10.1161/ATVBAHA.108.168690 18617650

[35] Cui L, Lee YH, Kumar Y, Xu F, Lu K, Ooi EE, et al Serum metabolome and lipidome changes in adult patients with primary dengue infection. PLoS neglected tropical diseases. 2013;7(8):e2373 10.1371/journal.pntd.0002373 23967362 PMC3744433

[36] Ekchariyawat P, Hamel R, Bernard E, Wichit S, Surasombatpattana P, Talignani L, et al Inflammasome signaling pathways exert antiviral effect against Chikungunya virus in human dermal fibroblasts. Infection, genetics and evolution: journal of molecular epidemiology and evolutionary genetics in infectious diseases. 2015;32:401–8. 10.1016/j.meegid.2015.03.025 25847693

[37] Chaturvedi UC, Elbishbishi EA, Agarwal R, Raghupathy R, Nagar R, Tandon R, et al Sequential production of cytokines by dengue virus-infected human peripheral blood leukocyte cultures. Journal of medical virology. 1999;59(3):335–40 10502266 10.1002/(sici)1096-9071(199911)59:3<335::aid-jmv13>3.0.co;2-e

[38] Azeredo EL, Zagne SM, Santiago MA, Gouvea AS, Santana AA, Neves-Souza PC, et al Characterisation of lymphocyte response and cytokine patterns in patients with dengue fever. Immunobiology. 2001;204(4):494–507. 10.1078/0171-2985-00058 11776403

[39] Tauseef A, Umar N, Sabir S, Akmal A, Sajjad S, Zulfiqar S. Interleukin-10 as a Marker of Disease Progression in Dengue Hemorrhagic Fever. Journal of the College of Physicians and Surgeons—Pakistan: JCPSP. 2016;26(3):187–90. doi: 03.2016/JCPSP.187190 26975948

[40] Suharti C, van Gorp EC, Dolmans WM, Setiati TE, Hack CE, Djokomoeljanto R, et al Cytokine patterns during dengue shock syndrome. European cytokine network. 2003;14(3):172–7 14656693

[41] Kuno G, Bailey RE. Cytokine responses to dengue infection among Puerto Rican patients. Memorias do Instituto Oswaldo Cruz. 1994;89(2):179–82 7885241 10.1590/s0074-02761994000200010

[42] Chen JP, Lu HL, Lai SL, Campanella GS, Sung JM, Lu MY, et al Dengue virus induces expression of CXC chemokine ligand 10/IFN-gamma-inducible protein 10, which competitively inhibits viral binding to cell surface heparan sulfate. J Immunol. 2006;177(5):3185–92 16920957 10.4049/jimmunol.177.5.3185

[43] Brasier AR, Ju H, Garcia J, Spratt HM, Victor SS, Forshey BM, et al A three-component biomarker panel for prediction of dengue hemorrhagic fever. The American journal of tropical medicine and hygiene. 2012;86(2):341–8. 10.4269/ajtmh.2012.11-0469 22302872 PMC3269290

[44] Eppy Suhendro, Nainggolan L, Rumende CM. The Differences Between Interleukin-6 and C-reactive Protein Levels Among Adult Patients of Dengue Infection with and without Plasma Leakage. Acta medica Indonesiana. 2016;48(1):3–9 27241538

[45] Liu W, Yin Y, Zhou Z, He M, Dai Y. OxLDL-induced IL-1 beta secretion promoting foam cells formation was mainly via CD36 mediated ROS production leading to NLRP3 inflammasome activation. Inflammation research: official journal of the European Histamine Research Society [et al]. 2014;63(1):33–43. 10.1007/s00011-013-0667-3 24121974

[46] Soto-Acosta R, Mosso C, Cervantes-Salazar M, Puerta-Guardo H, Medina F, Favari L, et al The increase in cholesterol levels at early stages after dengue virus infection correlates with an augment in LDL particle uptake and HMG-CoA reductase activity. Virology. 2013;442(2):132–47. 10.1016/j.virol.2013.04.003 23642566

[47] Kellner-Weibel G, Yancey PG, Jerome WG, Walser T, Mason RP, Phillips MC, et al Crystallization of free cholesterol in model macrophage foam cells. Arterioscler Thromb Vasc Biol. 1999;19(8):1891–8 10446067 10.1161/01.atv.19.8.1891

[48] Duewell P, Kono H, Rayner KJ, Sirois CM, Vladimer G, Bauernfeind FG, et al NLRP3 inflammasomes are required for atherogenesis and activated by cholesterol crystals. Nature. 2010;464(7293):1357–61. 10.1038/nature08938 20428172 PMC2946640

