# Supplementary figures and images for: Inflammatory status and severity of disease in dengue patients are associated with lipoprotein alterations
Source: PLoS One. 2019 Mar 22;14(3):e0214245. doi: 10.1371/journal.pone.0214245 (PMC6430398; doi:10.1371/journal.pone.0214245)

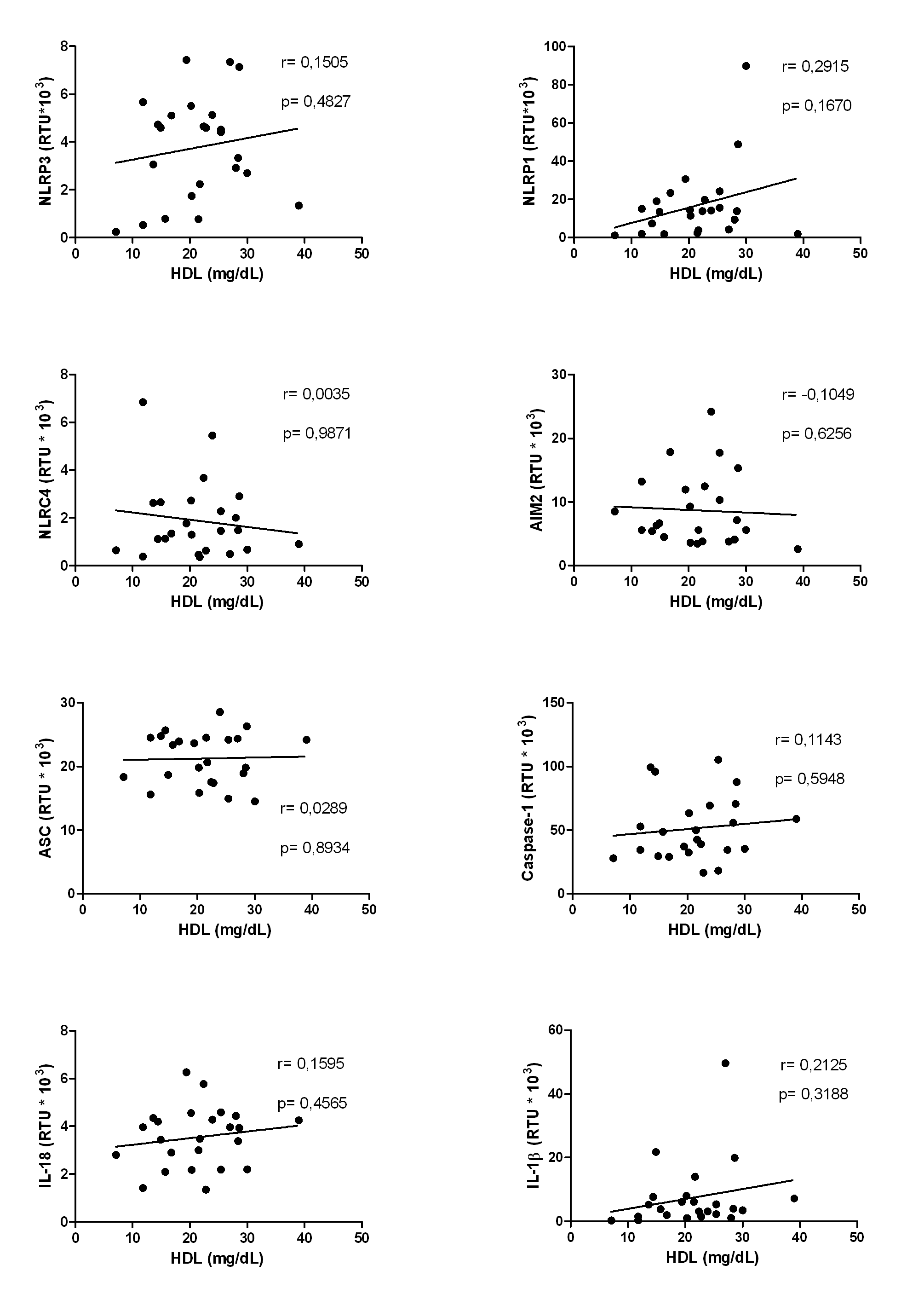

Supplement: S1 Fig — Correlation between HDL levels and (A) NLRP3, (B) NLRP1, (C) NLRC4, (D) AIM2, (E) Caspase-1, (F) ASC, (G) IL-18 and (H) IL-1β were assessed by the Spearman test. The r-value and p-value are indicated in the figure. A p-value lower than 0.05 was considered a significant correlation. (TIF) [file pone.0214245.s001.tif]
