# Supplementary material for: Inflammatory status and severity of disease in dengue patients are associated with lipoprotein alterations
Source: PLoS One. 2019 Mar 22;14(3):e0214245. doi: 10.1371/journal.pone.0214245 (PMC6430398; doi:10.1371/journal.pone.0214245)
Supplement: S1 Table — (DOCX) [file pone.0214245.s002.docx]

S1 Table. Sequence of the primers

| **Gen** | **Secuencia de primers 5´-3´** | **Temperatura de *annealing*** | **Longitud del producto (pb)** |
| --- | --- | --- | --- |
| **IL-1β** | Fw: GGATATGGAGCAACAAGTGG  Rv: ATGTACCAGTTGGGGAACTG | 60 °C | 264 |
| **IL-18** | Fw: ATGGCTGCTGAACCAGTAGAAG  Rv: CAGCCATACCTCTAGGCTGGC | 62 °C | 292 |
| **NLRP3** | Fw: AGCACCAGCCAGAGTCTAAC  Rv: CCCCAACCACAATCTCCGAAT | 57 °C | 123 |
| **NLRP1** | Fw: ACTATACTTCCCGAGGCATCCTT  Rv: TGGTCTTGGAAGTCAGTGTGAGT | 56 °C | 301 |
| **NLRC4** | Fw: CTCTCATGGTGGAAGCCAGTCC  Rv: GACAGAGACTTGACTATGTAATCC | 56 °C | 301 |
| **AIM2** | Fw: AAGCGCTGTTTGCCAGTTAT  Rv: CACACGTGAGGCGCTATTTA | 55 °C | 231 |
| **ASC** | Fw: AACCCAAGCAAGATGCGGAAG  Rv: TTAGGGCCTGGAGGAGCAAG | 62 °C | 82 |
| **Caspasa-1** | Fw: CAAGGGTGCTGAACAAGG  Rv: GGGCATAGCTGGGTTGTC | 60 °C | 278 |
| **B-actina** | Fw: CTTTGCCGATCCGCCGC  Rv: ATCACGCCCTGGTGCCTGG | 60 °C | 174 |
